# Ca^2+^ Efflux Is Involved in Cinnamaldehyde-Induced Growth Inhibition of *Phytophthora capsici*

**DOI:** 10.1371/journal.pone.0076264

**Published:** 2013-10-01

**Authors:** Liangbin Hu, Dede Wang, Li Liu, Jian Chen, Yanfeng Xue, Zhiqi Shi

**Affiliations:** 1 Institute of Food Safety and Monitoring Technology, Jiangsu Academy of Agricultural Sciences, Nanjing, China; 2 Plant Protect College, Nanjing Agricultural University, Nanjing, China; 3 School of Life Science, Nanjing Normal University, Nanjing, China; 4 School of Food Science, Henan Institute of Science and Technology, Xinxiang, China; Woosuk University, Korea, Republic Of

## Abstract

As a destructive fungus-like plant pathogen, the oomycete *Phytophthoracapsici* is unable to synthesize its own ergosterol as the potential target of fungicide cinnamaldehyde (CA). In this study, CA exerted efficient inhibitory effects on both mycelial growth (EC50=0.75 mM) and zoospore germination (MIC=0.4 mM) of *P* *. capsici* . CA-induced immediate Ca^2+^ efflux from zoospores could be confirmed by the rapid decrease in intracellular Ca^2+^ content determined by using Fluo-3 AM and the increase in extracellular Ca^2+^ concentration determined by using ICP-AES (inductively coupled plasma atomic emission spectrometry). Blocking Ca^2+^ influx with ruthenium red and verapamil led to a higher level of CA-induced Ca^2+^ efflux, suggesting the simultaneous occurrence of Ca^2+^ influx along with the Ca^2+^ efflux under CA exposure. Further results showed that EGTA-induced decrease in intracellular Ca^2+^ gave rise to the impaired vitality of *P* *. capsici* while the addition of exogenous Ca^2+^ could suppress the growth inhibitory effect of CA. These results suggested that Ca^2+^ efflux played an important role in CA-induced growth inhibition of *P* *. capsici* . The application of 3-phenyl-1-propanal, a CA analog without α,β- unsaturated bond, resulted in a marked Ca^2+^ influx in zoospores but did not show any growth inhibitory effects. In addition, exogenous cysteine, an antagonist against the Michael addition (the nucleophilic addition of a carbanion or another nucleophile) between CA and its targets, could attenuate CA-induced growth inhibition of *P* *. capsici* by suppressing Ca^2+^ efflux. Our results suggest that CA inhibits the growth of *P* *. capsici* by stimulating a transient Ca^2+^ efflux via Michael addition, which provides important new insights into the antimicrobial action of CA.

## Introduction

The oomycete Phytophthora *capsici* (Leonian) is a destructive fungus-like plant pathogen, which infects solanaceous and cucurbitaceous hosts including snap, lima, cucumber, eggplant, tomato, pepper, pumpkin, squash, melon, and zucchini [1]. *P* *. capsici* has both a sexual and asexual life of cycle. Plants infected with *P* *. capsici* show various disease symptoms, such as foliar blights, fruit rots, stem and root rots [2]. The preventive and frequent application of fungicides can limit disease expanding [3], but the increasing resistance of *P* *. capsici* to fungicides such as mefenoxam and pyrimorph, has been widely documented in some vegetable production regions [1,4]. New fungicides aiming at different targets need to be timely developed to overcome this resistance.

Cinnamaldehyde (CA), a major constituent of cinnamon essential oils, exists naturally in the bark and leaves of cinnamon trees of the genus *Cinnamomum* [5]. CA has been developed as food antimicrobial agent due to its activity against bacteria, yeast, and filamentous molds [6,7]. So far, available experimental evidence suggests that antimicrobial action of CA involves cell wall synthesis, membrane action, and specific cellular processes [6-9]. However, the defined targets of cinnamaldehyde in microbial have not well established yet, which requires more investigations in the early microbial responses to CA.

Ca^2+^ is not only a universal intracellular second messenger in eukaryotic cells, but also is essential for multiple functions of cell compartments [10]. In fungi, Ca^2+^ regulates numerous intrinsic metabolic processes, such as spore germination, tip growth, hyphal branching, sporulation, infection structure differentiation, and circadian clocks, as well as responses to various environmental stress [11-16]. The disruption of Ca^2+^ signaling/homeostasis is able to result in the inhibition of some fugal growth [16-18]. However, the link between the fungicidal activity of CA and the disturbance of Ca^2+^ homeostasis has not been established yet. Mammalian TRP (Transient Receptor Potential) are nonselective cation-permeable channels, most of which are permeable for Ca^2+^ [19]. In mouse cells, CA activates TRPA1 covalently binding, leading to a Ca^2+^ influx [20,21]. Whether the disruption of intracellular Ca^2+^ is involved in the fungicide action of CA remains to be investigated. In this study, we found that CA could efficiently inhibit the growth of *P* *. capsici* by stimulating an immediately Ca^2+^ efflux in vivo. The results provide important new insights into the fungicidal action of CA.

## Materials and Methods

### Strain and Culture conditions

*P* *. capsici* strain (Institution of plant protection, Nanjing Agricultural University) was maintained on potato dextrose agar (PDA) medium at 28°C. For liquid cultures, *P* *. capsici* strains were grown in Potato Dextrose (PD) medium at 28°C at 100 rpm.

### Induction of *P. capsici* Zoospore

A zoospore suspension was obtained according to Silvar et al. [22]. *P* *. capsici* isolate was grown in PDA medium at 24°C for 7 days. PDA cultures were cut into small pieces and incubated with V8 broth (160 ml of clarified V8 juice cleared by centrifuging V8 juice amended with 1.6 g of CaCO_3_/l, 4000 rpm/min, 10 min, then added deionized water to 1000 ml) in dark at 24°C for 2 days. Then these small pieces were transferred into sterile distilled water (SDW) and incubated in light at 24°C for 3 days. Zoospore release was induced by chilling cultures at 4°C for 30 min and then incubating at room temperature for 30-60 min. Zoospore was separated from the empty sporangia by passing the liquid through a four-layer facial tissue and was induced to encyst by vortexing for 5 min. Concentration of zoospore was adjusted to 10^5^ zoospores/ml. Zoospore concentration was counted using a haemocytometer.

### Mycelial Growth and Zoospore Germination

The influence of CA (Aladdin, China) on mycelial growth of *P* *. capsici* was determined at 28°C on potato dextrose agar (PDA) medium adjusted to different concentrations of CA. Mycelial discs (6 mm in diameter) of *P* *. capsici* grown on PDA plates were cut from the margins of 7-days-old colony and transferred to the center of PDA plates containing different concentrations of CA (0, 0.2, 0.4, 0.6, 0.8, 1.0, 1.5, 2.0 mM). After incubation at 28°C for 2 days, mycelial radial growth was measured for calculating EC_50_ (the concentration inhibiting growth by 50%) with the Data Processing System (DPS) (Hangzhou Reifeng Information Technology) [23]. Effects of CA on zoospore germination and growth were tested in 96 well microtiter plate (MaxiSorp Nunc). Each well contained 180 µl of PD medium (1×10^5^ zoospores/ml), and then 20 µl of CA solution (mixed in PD medium) was added into these wells to the final concentrations of 0 (control), 0.1, 0.2, 0.3, 0.4, 0.5 and 0.6 mM, respectively. The plate was incubated at 28°C, and zoospore germination was assessed through microscopic observation with an in-verted microscope (ECLIPSE, TE2000-S, Nikon) when all the zoospores in the control well had just almost germinated. After that, the plate was incubated for 2 days for the determination of MIC (the lowest concentration showing complete inhibition of visible mycelial formation). In addition, optical density at 600 nm (OD600) of these wells was also determined to point out the effects of the other additions on the inhibitory activity of CA to *P* *. capsici* .

### Cell viability

Fungal cell viability was determined by the percent reduction of Alamar Blue (Invitrogen, USA) using a Alamar Bluecell viability assay kit (Biotium, Inc.) When added to cell cultures, the oxidized form of the Alamar Blue (AB) is converted to the reduced form by accepting electrons from NADPH leading to the colour of the culture medium transformed to pink. The AB assay reagent was added into the well of a 96-well plate containing 100 µl of zoospore suspension (1×10^5^ cells/ml) in PD medium with the addition of EGTA at given concentrations. Subsequently, the plate was incubated at 28°C for 6h in the dark, and the medium color of each test well was observed and photographed.

### Measuring [Ca ^2+^] Levels in vivo

To test the changes of *P* *. capsici* zoospores cellular calcium ion with CA treatment, the intracellular concentration of free Ca^2+^ was monitored using the Ca^2+^-binding probe Fluo-3 AM (Beyotime, China). As a membrane-permeable and non-ratiometric dye, Fluo-3 AM can be cut into Fluo-3 after enters cell and bind cytosolic free Ca^2+^. Fluo-3 AM was dissolved in DMSO in a 5 mM stock solution. The ultimate Fluo-3 fluorescence were recorded using wavelength setting of excitation at 506 nm and emission at 526 nm upon Ca^2+^ binding [24]. Fluo-3 AM was added into the well of a 96-well plate which contained 100 µl PD medium (1×10^5^ zoospores/ml), and then the inoculum was incubated at 28°C for 30 min in the dark. Given amount of CA was added into the well every 8 min and its concentration arrived at a value increasing from 0 to 2.0 mM after addition of 6 times. During the whole course, fluorescence of each independent well was continuously monitored with a measure frequency of 20 s using a fluorescence plate reader (Thermo Fischer, United Kingdom). The continuous changes of calcium-dependent fluorescence for each trace were calculated use relative fluorescence units (RFU). A representative trace of three repeats of each experiment was shown.

### Measuring Released [Ca ^2+^] Levels in vitro

Released Ca^2+^ from zoospores was determined using inductively coupled plasma atomic emission spectrometry (ICP-AES, Optima 2100DV, PE, USA). Twenty milliliters of zoospore suspension of *P* *. capsici* (1×10^5^ zoospores/ml) passed through a 0.22 µm filter (Supor, Pall Life Sciences), and then the filter was eluted with 0.01 mM EDTA (50 ml) to eliminate the divalent cations. Undergoing another elution with 100 ml distilled water, the zoospores were collected from the film and suspended in distilled water to a final concentration of 1×10^5^ zoospores/ml. The new zoospore suspension was immediately exposed to 0.4 mM CA for 8 min (no CA for the control), and then passed through the filter. The filtrate was concentrated by 10 fold through rotary vacuum evaporation. The ICP-AES was performed to detect Ca^2+^ contentration in the filtrate.

### Statistics

All the data presented are mean value ± standard errors of the means (SEM) of three determinations. Student’s *t*-test was used to determine significant differences between means in all experiments. The differences were considered significant at P < 0.05.

## Results

### Inhibitory effects of CA on *P. capsici* growth

**Figure 1:**
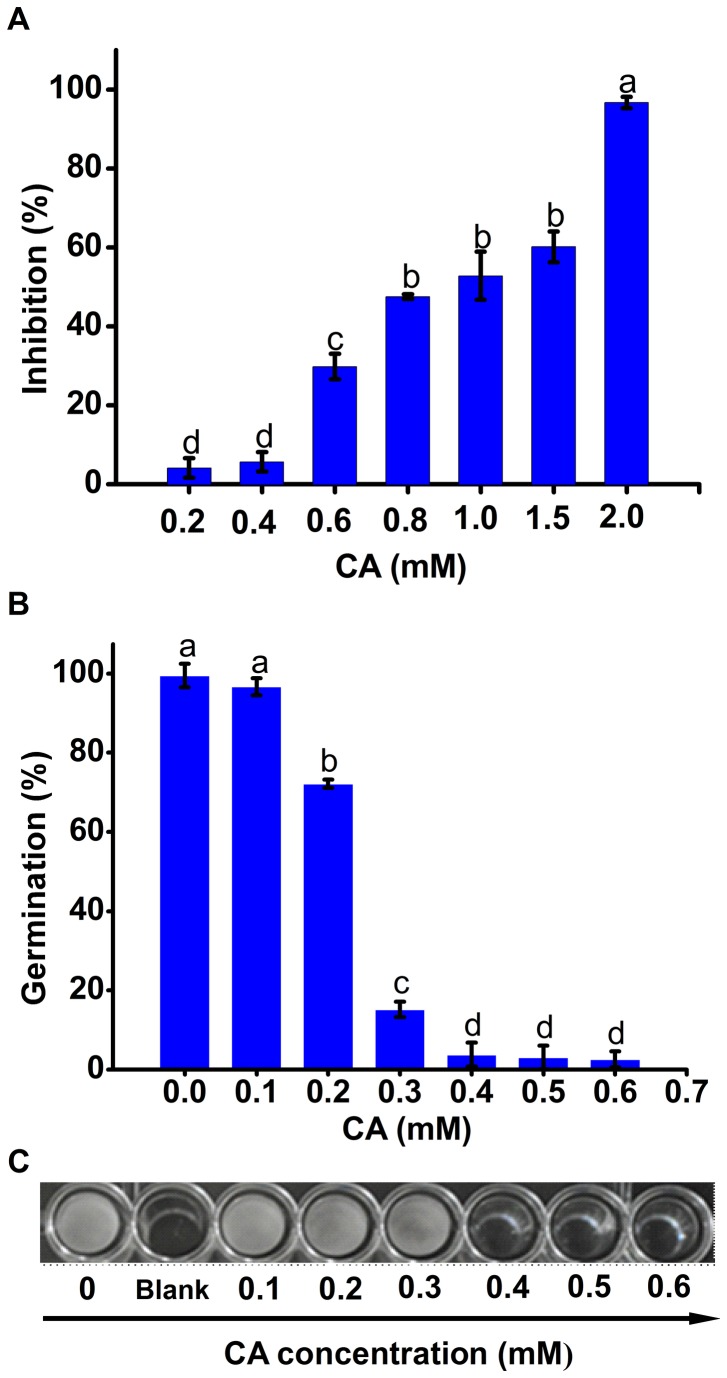
Inhibitory effects of CA on the growth of *P* *. capsici* . (A) Radial mycelial growth of *P* *. capsici* with CA treatment. (B) Germination rate of zoospores with CA treatment. (C) Growth of zoospores exposed to CA in 96-well-plate for 48 h; the control well (0 mM CA) was insulated from other wells containing CA by a blank well. Each bar indicated the means of three replicates ± standard error. Different letter indicate a significant difference between them (P <0.05).

CA showed inhibitory effects on both mycelial radical elongation and zoospore germination (Figure 1). According to the dose-dependent inhibition of CA on mycelial growth of *P* *. capsici* (Figure 1A), the EC50 value was calculated as approximately 0.75 mM. However, the concentration of CA needed to arrive at more than 2 mM to completely inhibit mycelial growth (Figure 1A). Compared with mycelia, zoospores of *P* *. capsici* were more sensitive to CA. Zoospore germination could be significantly decreased in the presence of 0.2 mM of CA. Few zoospores could germinate normally from an exposure to more than 0.4 mM CA (Figure 1B). The formation of mycelia was almost completely inhibited under the treatment of 0.4 mM CA for 48 h (Figure 1C). Taken together, we defined the MIC value of CA against zoospore germination and growth was 0.4 mM.

### CA induced immediate Ca^2+^ efflux from zoospores

**Figure 2:**
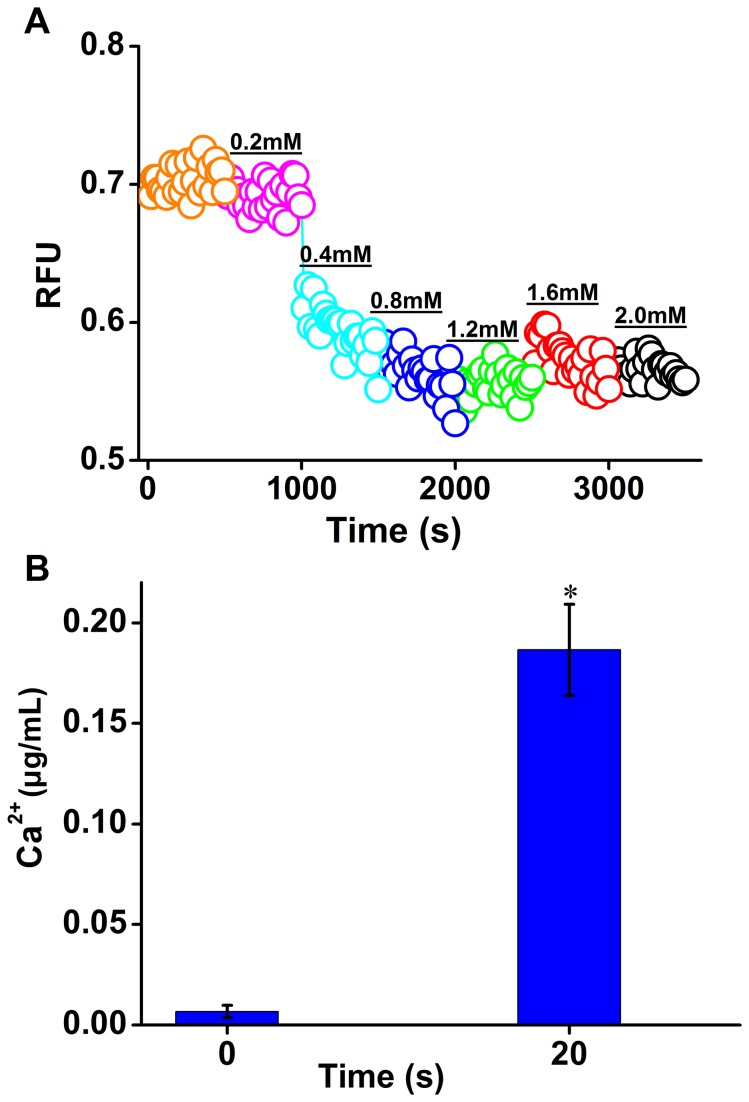
CA induced immediate Ca^2+^ efflux in zoospores of *P* *. capsici* . (A) Free Ca^2+^ content changes in Zoospores of *P* *. capsici* with CA treatment. A quantity of CA was added into the well every 8 min and its concentration arrived at 0.2, 0.4, 0.8, 1.2, 1.6, and 2.0 mM respectively. Fluo-3 AM was utilized to monitor the intracellular free Ca^2+^. Fluorescence was monitored with a measure frequency 20 s using a fluorescence plate reader. A representative trace of three repeats of each experiment was shown. (B) Extracellular Ca^2+^ increase in the zoospore suspension under stimulation with CA. The content of extracellular Ca^2+^ was determined using ICP-AES. The bar indicated the means of three replicates ± standard error. *, a significant increase of extracellular Ca^2+^ 20 s post the exposure to CA compared to that before the addition of CA (P<0.05).

CA has been showed to activate TRP of mammalian cells and lead to a Ca^2+^ influx. To examine the effect of CA on free Ca^2+^ levels in zoospores of *P* *. capsici* , the calcium-sensitive fluorescent dye Fluo-3 AM was applied as an indicator of free intracellular Ca^2+^. It was surprising to find that exposure to CA resulted in an immediate decrease in Fluo-3 fluorescence intensity (RFU) (Figure 2A), which indicated a decline of free Ca^2+^ level in zoospores. The remarkable decrease in RFU occurred just when 0.4 mM of CA was added (Figure 2A). Subsequently free Ca^2+^ level in zoospores tended to be stable even with continual addition of CA (Figure 2A). The decreased free Ca^2+^ level in zoospores cannot equal to the increased extracellular free Ca^2+^ level because Ca^2+^ can also be stored into calcium pool [25]. To monitor the destiny of decreased Ca^2+^, we determined the changes in the extracellular free Ca^2+^ level in response to 0.4 mM of CA. The extracellular free Ca^2+^ level exhibited a significant increase with an exposure to CA for 20 s (P<0.05) (Figure 2B), which confirmed the existence of Ca^2+^ efflux to extracellular space in zoospores of *P* *. capsici* exposed to CA.

### Simultaneous Ca^2+^ influx in zoospores

**Figure 3:**
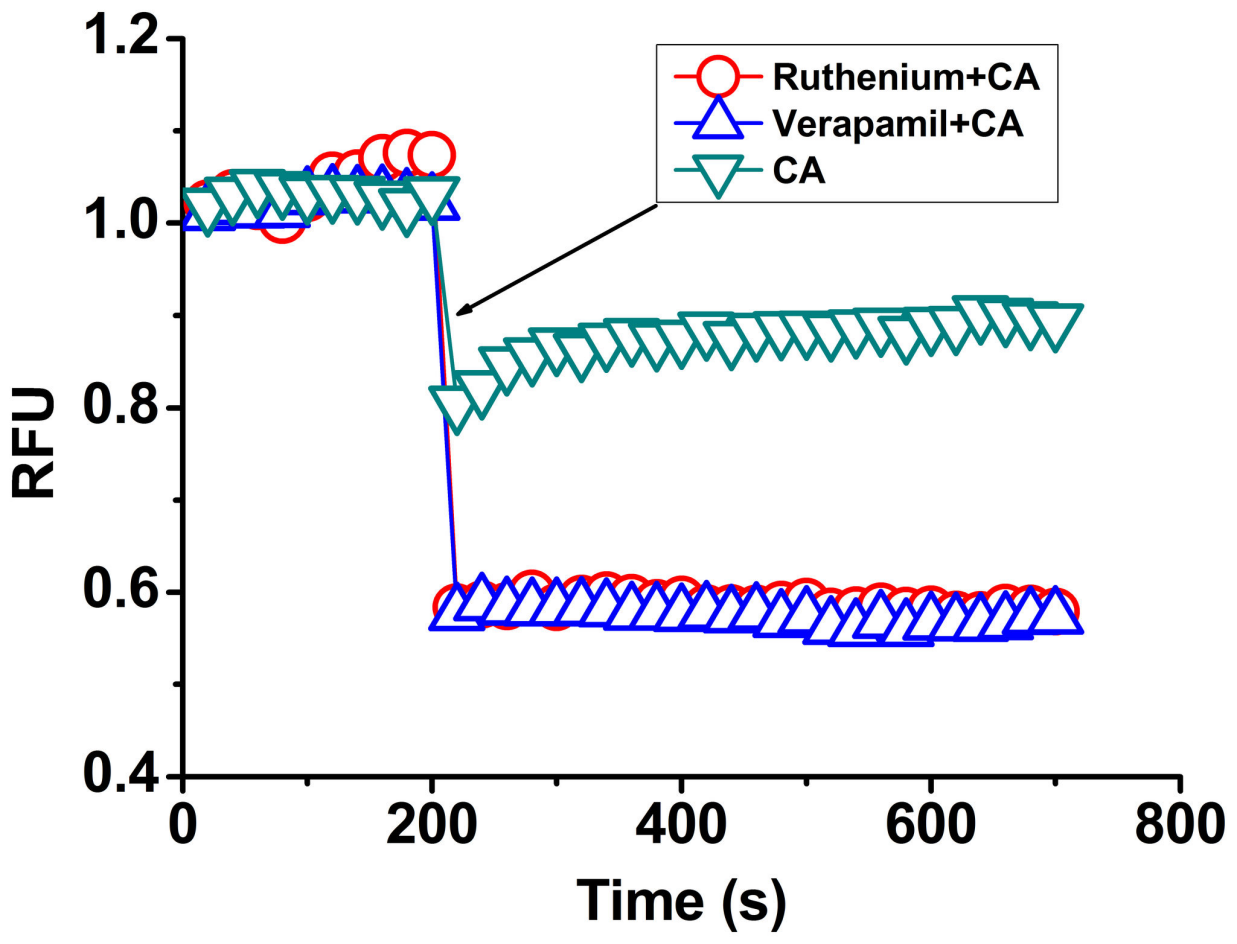
Changes of CA-induced Ca^2+^ efflux in response to the addition of some inhibitors associated with Ca^2+^ flux. All tested agents were added post incubation at 28°C for 200 s; ▽, 0.4 mM CA; Δ, 5 µg/mL Ruthenium red + 0.4 mM CA; ○, 5 µg/mL Verapamil + 0.4 mM CA. Verapamil and Ruthenium red, voltage-dependent calcium channel blockers. A representative trace of three repeats of each experiment was shown.

In order to study CA-induced disturbance of Ca^2+^ homeostasis in detail, we also investigated the effect of CA on Ca^2+^ influx in zoospores. The addition of ruthenium red and verapamil, two voltage-dependent calcium channel blockers [26], led to rapid decreases in intracellular Ca^2+^ level in zoospores indicated with Fluo-3 fluorescence (Figure 3). This result suggested that Ca^2+^ influx occurred simultaneously in response to the loss of Ca^2+^ due to Ca^2+^ efflux in the action of CA. However, the higher Ca^2+^ efflux than influx may resulted in the final loss of intracellular free Ca^2+^ in zoospores.

### CA-induced Ca^2+^ efflux contributed to the growth inhibition of zoospores

**Figure 4:**
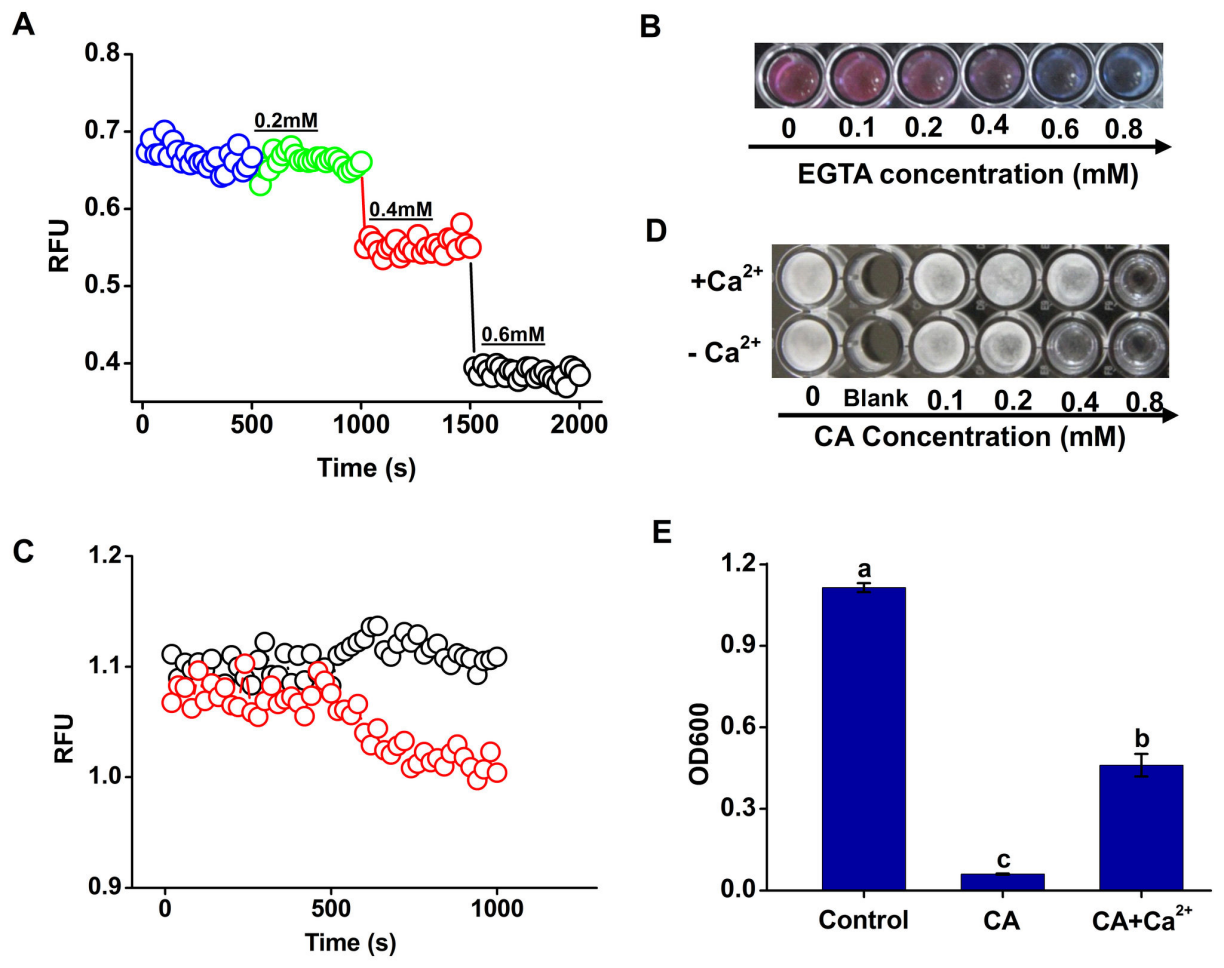
Involvement of Ca^2+^ efflux in the growth inhibition of *P* *. capsici* zoospores by CA. (A) Effects of Specific Ca^2+^ chelant EGTA on the the cytosolic Ca^2+^ content. A quantity of EGTA was added into the well every 8 min and its concentration arrived at 0.2, 0.4, and 0.6 mM respectively. A representative trace of three repeats of each experiment was shown. (B) Zoospore vitality of *P* *. capsici* exposed to EGTA at the given concentrations for 8 h. (C) Changes of CA-induced Ca^2+^ efflux in response to the addition of exogenous Ca^2+^. Red “○”, only CA was added at 8 min post incubation to a final concentration of 0.4 mM respectively; Black “○”, CaCl_2_ was added to a final concentration of 1.5 mM before the addition of CA at 8 min post incubation, and then the following operation was the same to the former. A representative trace of three repeats of each experiment was shown. (D) Mycelial formation from zoospores exposed to CA and CA+Ca^2+^ for 48 h. The results shown were obtained from one of three independent experiments. (E) Optical density at 600 nm (OD600) resulted from the growth of *P* *. capsici* . The addition of Ca^2+^ significantly reduced the growth inhibition of *P* *. capsici* by CA (P<0.05).

The intracellular Ca^2+^ homeostasis is very important to regular fugal metabolism. To test the role of Ca^2+^ efflux in the inhibition by CA, the free intracellular Ca^2+^ level was decreased by the addition of EGTA (a specific Ca^2+^ chelator). The results showed 0.4 mM of EGTA induced the decreases in the intracellular Ca^2+^ level and the zoospore vitality (Figure 4A and B), suggesting that the intracellular free Ca^2+^ is essential for the growth of *P* *. capsici* . Next, we tested whether exogenous supplement of Ca^2+^ could reverse CA-induced inhibitory effect on *P* *. capsici* . The addition of exogenous Ca^2+^ significantly enhanced intracellular Ca^2+^ level in CA-treated zoospores (Figure 4C), and also stimulated the mycelial formation from zoospores under CA treatments (Figure 4D). Compared to the treatment of CA (0.4 mM) alone, the growth of *P* *. capsici* represented by OD600 increased by 7.7 fold under the simultaneous treatment with 1.5 mM of Ca^2+^ (Figure 4E). These results suggested that Ca^2+^ efflux contributed to CA-induced growth inhibition of *P* *. capsici* zoospores.

### CA Induced Ca^2+^ efflux and growth inhibition of *P. capsici* through Michael addition

**Figure 5:**
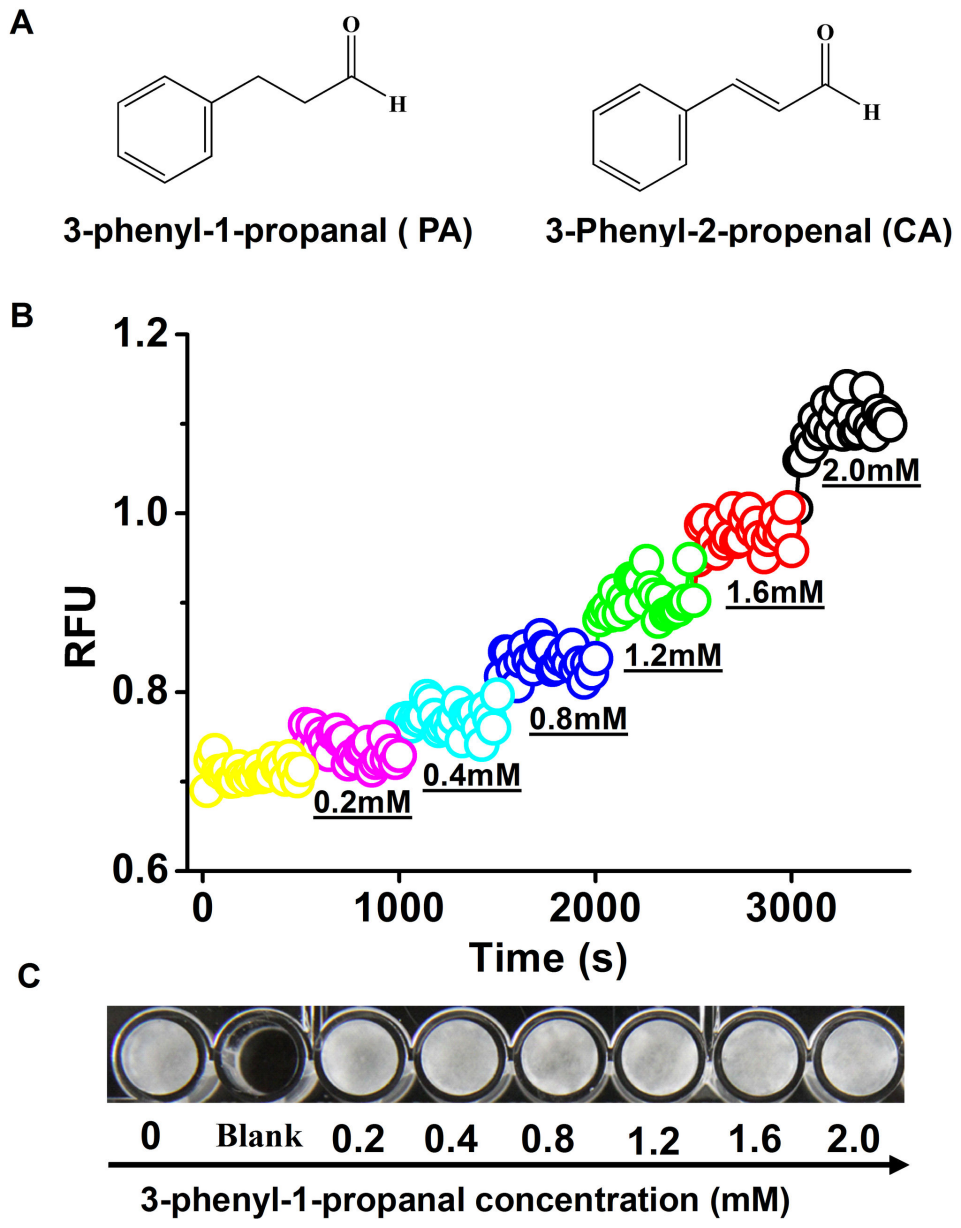
Effects of 3-phenyl-1-propanal (PA, a CA analog) (A) on the Ca^2+^ content (B) and growth (C) of *P* *. capsici* zoospores. (A) Chemical structure formulas of 3-phenyl-1-propanal and CA. (B) A quantity of 3-phenyl-1-propanal was added into the well every 8 min and its concentration arrived at 0.2, 0.4, 0.8, 1.2, 1.6, and 2.0 mM respectively. (C) Zoospore growth of *P. capsic*i exposed to 3-phenyl-1-propanal at the given concentrations for 48 h. A representative trace of three repeats of each experiment was shown.

**Figure 6:**
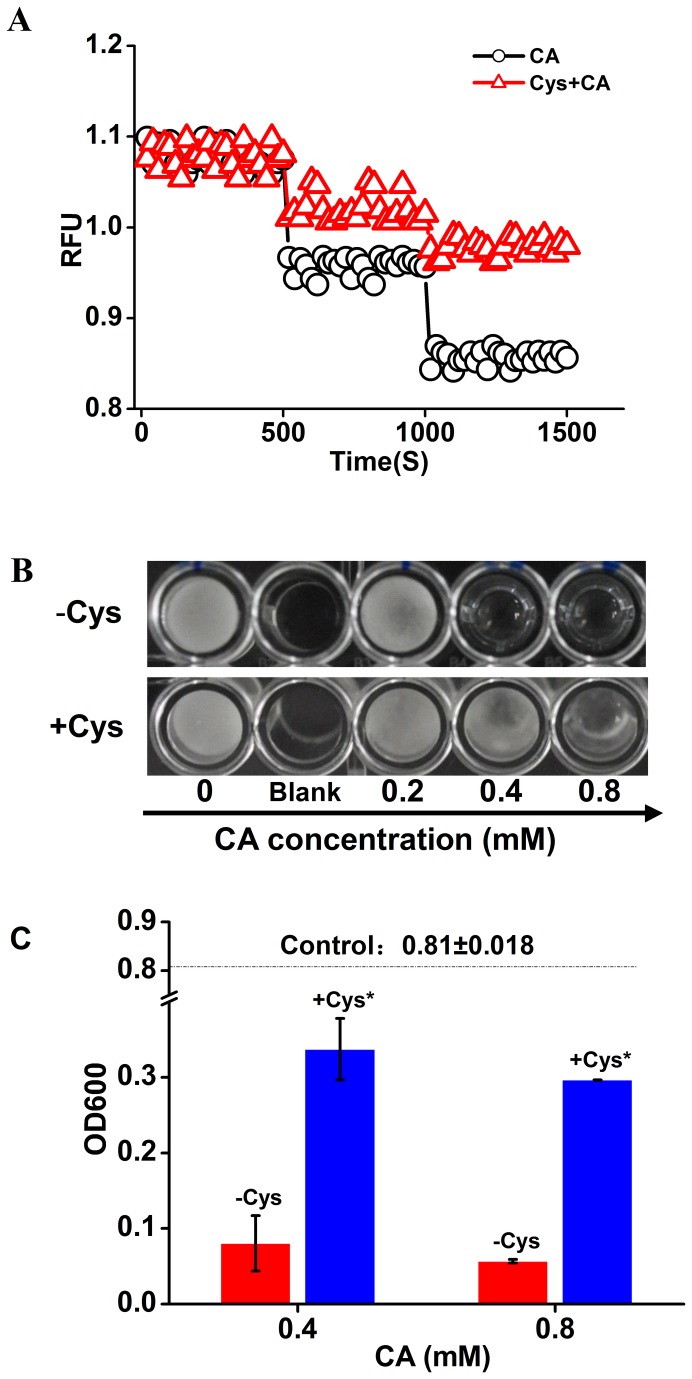
Changes of CA-induced Ca^2+^ efflux and growth inhibition of zoospores in response to the addition of cysteine. (A) Δ, only CA was added at 8 min and 16 min post incubation at 28°C to a final concentration of 0.4 and 0.8 mM respectively; ○, cysteine (Cys) was added to a final concentration of 1.5 mM before the addition of CA at 8 min post incubation, and then the following operation was the same to the former. A representative trace of three repeats of each experiment was shown. (B) Mycelial formation from zoospores exposed to CA and CA+Cys at the indicated concentrations for 48 h. The results shown were obtained from one of three independent experiments. (C) Optical density at 600 nm resulted from the growth of *P* *. capsici* . The dotted line indicated OD600 of control (without CA). *, the addition of Cys significantly reduced the growth inhibition of *P* *. capsici* by CA (P<0.05).

The nucleophilic mercapto group cysteines of TRP can attack the α,β-unsaturated bond of (CA) via a Michael addition leading to a Ca^2+^ influx, which suggests that the α,β-unsaturated bond is essential for CA targeting to TRP [21]. To understand the involvement of Michael addition in CA-induced Ca^2+^ efflux, we monitored the changes of intracellular Ca^2+^ level in zoospores exposed to 3-phenyl-1-propanal (PA, a CA analog without α,β-unsaturated bond) (Figure 5A and B). Treatment with PA led to a Ca^2+^ influx but not efflux in zoospores with a dose-dependent manner (Figure 5 B). CA at 0.4 mM significantly inhibited the growth of zoospores (Figure 1), but PA did not show any inhibitory effects on the growth of zoospores even up to the concentration of 2 mM (Figure 5C). In addition, exogenous cysteine was utilized as an antagonist against the addition between CA and its potential targets. Treatment with 1.5 mM of cysteine was able to suppress the Ca^2+^ efflux in zoospores exposed to CA at the concentration of 0.4 mM and 0.8 mM (Figure 6A). The parallel antimicrobial assay indicated that the addition of cysteine significantly antagonized CA-induced growth inhibition of *P* *. capsici* zoospores (Figure 6B and C). These results indicated that Michael addition to α,β-unsaturated bond of CA was very important for stimulating Ca^2+^ efflux and the subsequent growth inhibition of *P* *. capsici* *.*

## Discussion

Our results showed CA could efficiently inhibit the growth of *P* *. capsici* . Due to its quality belonging to food additives, CA could be a safe alternative in the control of agricultural diseases caused by *P* *. capsici* . The antifungal activity of CA has been attributed to its significant effect on ergosterol production [7]. However, *Phytophthora* species are unable to synthesize their own sterols [27], which suggested a new mechanism involving the inhibition of *P* *. capsici* by CA. The results derived from chemical structure-antifungal activity relationship of CA suggests that the aromatic ring and the length of hydrocarbon chain outside the ring of CA affect its antifungal properties [28]. However, either hydrogenation of α,β-unsaturated carbonyl moiety of CA (PA), or the addition with some antagonists of Michael addition, led to a severally suppressed inhibitory effects on the growth of *P* *. capsici* . These results revealed that the α,β-unsaturated carbonyl moiety of CA played a vital role in its anti-*P. capsici* action via Michael additions.

CA is capable of stimulating a Ca^2+^ influx by activating TRPA1 ion channels via a Michael reaction [19]. Intracellular Ca^2+^ distribution is tightly controlled by active (ATP-dependent) membrane calcium pumps. Several types of perturbations in Ca^2+^ homeostasis will elicit cell death [29,30]. Calcium homeostasis has proven as the action site of some antifungal agents [16,18], In the present study, four lines of evidence indicated that CA-disturbed Ca^2+^ homeostasis was involved in CA-induced growth inhibition of *P* *. capsici* . Firstly, CA-induced inhibition of the growth of *P* *. capsici* was accompanied with the increase in a rapid Ca^2+^ efflux in zoospores. Secondly, the addition of Ca^2+^ chelator EGTA resulted in the decreases in the intracellular Ca^2+^ level and the vitality of zoospores. Thirdly, the addition of exogenous Ca^2+^ not only compromised the decrease of intracellular Ca^2+^ level induced by CA but also significantly ameliorated CA-induced growth inhibition of *P* *. capsici* . Fourthly, treatment with PA (a CA analog without α,β-unsaturated bond) failed to induce Ca^2+^ efflux and growth inhibition of zoospores.

It is still unclear whether the state of Ca^2+^ level stored in cytoplasm or the pathway involving disturbance of calcium homeostasis triggers the fungal growth inhibition. Our results indicated Ca^2+^ influx induced by PA instead of CA did not lead to the growth inhibition of *P* *. capsici* . Thus it could be concluded that not all the disturbance to calcium homeostasis are fatal to the fungal growth, and the pathway should be especially concerned in the association between calcium homeostasis and antifungal action. Calcium efflux has been showed associated with broad-based fungicidal activity of amiodarone [31]. Calcium efflux was also highlighted in the growth inhibition of *P* *. capsici* by CA. So far, the plasma membrane is revealed containing two systems for Ca^2+^ efflux: Na/Ca^2+^ exchanger (NCX) and Ca^2+^-ATPase (the plasma membrane Ca^2+^ pump, PMCA) [32]. Among them, only PMCA is identified in the fungal plasma membrane [33,34].

However, there is a Ca^2+^/H^+^ Exchanger (CAX) exporting Ca^2+^ of the cytosol to maintain optimal ionic concentrations in fungi cell [35]. Based on the research results from genomic-annotations of *P* *. capsici* (http://genome.jgi-psf.org/Phyca11/Phyca11.home.html), there were 16 potential Ca^2+^- pumps (Ca^2+^-ATPases), while none of CAX homologue could be found in the present genomic data of *P* *. capsici* . PMCA has been showed associated with inhibition of NFκB nuclear translocation and to promote cell death by regulating calcium signaling [36]. Taken together, the new pattern involving the antifungal action of CA could be elucidated that CA stimulate a transient Ca^2+^ efflux via Michael additions with PMCAs of *P* *. capsici* , finally leading to its growth inhibition. Further research is needed to construct a platform for the independent expression of these Ca^2+^-ATPases and to determine the effects of CA on these ATPases.
